# Meta-analysis of pulse pressure variation (PPV) and stroke volume variation (SVV) studies: a few rotten apples can spoil the whole barrel

**DOI:** 10.1186/s13054-023-04765-3

**Published:** 2023-12-07

**Authors:** Frederic Michard, Denis Chemla, Jean-Louis Teboul

**Affiliations:** 1 MiCo, Vallamand, Switzerland; 2 Faculté de Médecine Paris-Saclay, Le Kremlin-Bicêtre, France

**Keywords:** Anaesthesia, Hemodynamic monitoring, Fluid responsiveness, Pulse pressure variation, Prediction

We read the meta-analysis by Messina et al. [1] on the effectiveness of pulse pressure variation (PPV) and stroke volume variation (SVV) in predicting fluid responsiveness with both interest and skepticism.

This meta-analysis included studies involving patients with an open chest, ventilated with a low tidal volume, or undergoing laparoscopic surgery (with increased abdominal pressure due to pneumoperitoneum). In these conditions, PPV and SVV are known to be unreliable to predict fluid responsiveness [2]. Including studies where these limitations are not respected inevitably leads to an overall moderate predictive value. In other words, it is entirely foreseeable that the performance of a diagnostic tool will be moderate when one fails to consider the well-known limitations associated with its use.

We appreciate the fact that the predictive value of PPV and SVV was assessed in various subgroups. Regrettably, it was not evaluated in the subgroup of patients meeting all the conditions conducive to the reliable use of PPV and SVV. What was the area under the curve (AUC) in the subgroup of patients undergoing non-laparoscopic surgery with a closed chest and a tidal volume of 7–9 ml/kg? Would it support the conclusion that PPV and SVV are only moderately accurate to predict fluid responsiveness?

We firmly believe that both PPV and SVV serve as reliable predictors of fluid responsiveness, provided that physiologic limitations to their use are respected [3, 4]. Since the initial description of PPV almost 25 yrs ago [5], these limitations have been extensively discussed in numerous articles, including in this journal [2], and are summarized once more in the “PPV-meter” shown in Fig. 1. Many of these limitations (e.g. atrial fibrillation, spontaneous breathing activity, low tidal volume) are encountered less frequently in patients undergoing major surgery with general anesthesia than in critically ill patients. Of note, a tidal volume of 7–9 ml/kg, commonly used during surgery [6], has been deemed safe [7]. For patients ventilated with a tidal volume < 7 ml/kg, the assessment of changes in PPV during a mini-fluid challenge [8] or a transient rise in tidal volume (aka tidal volume challenge) [9] has proved useful to predict fluid responsiveness. Unfortunately, these points were not addressed in the paper by Messina et al. [1].

**Fig. 1:**
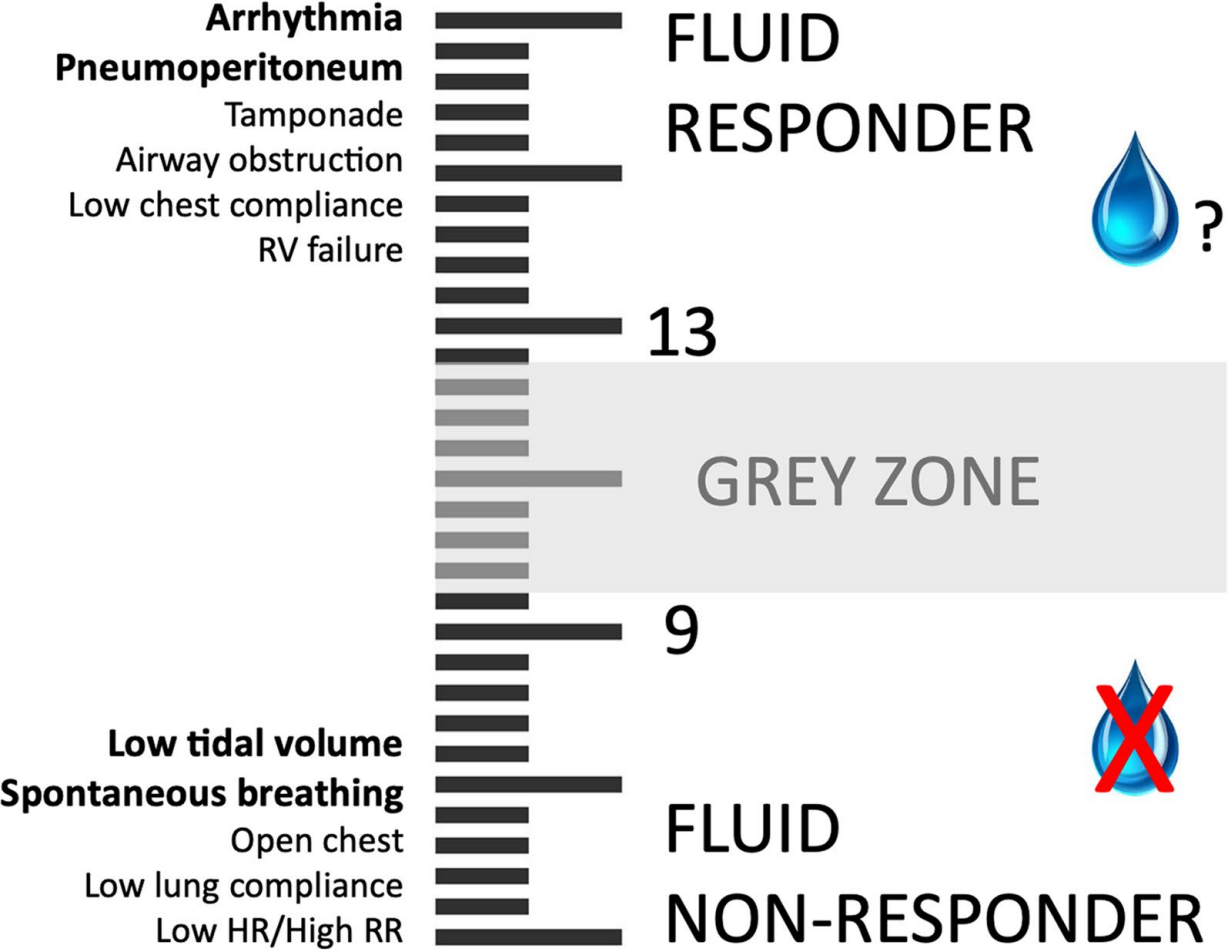
Pulse pressure variation (PPV)-meter summarizing the clinical meaning of PPV (right) and main limitations to its clinical use (left)

Finally, unlike SVV monitoring, PPV monitoring does not require any cardiac output monitoring device. In a meta-analysis assessing the respective performance of PPV and SVV, it would have been wise to highlight this practical advantage as well.

In summary, physiologic limitations to the use of PPV and SVV should be respected not only in clinical practice but also in meta-analyses; otherwise, they may lead to misleading conclusions. When these limitations are respected, we believe that both PPV and SVV are valuable variables for predicting fluid responsiveness and personalizing hemodynamic management, potentially leading to improved patient outcomes [10].

## Data Availability

Not applicable.

## Abbreviations

PPV: Pulse pressure variation
SVV: Stroke volume variation
AUC: Area under the curve

## References

[1] Messina A, Caporale M, Calabro L (2023). Reliability of pulse pressure and stroke volume variation in assessing fluid responsiveness in the operating room: a metaanalysis and a metaregression. Crit Care.

[2] Michard F, Chemla D, Teboul JL (2015). Applicability of pulse pressure variation: how many shades of grey?. Crit Care.

[3] Michard F (2005). Changes in arterial pressure during mechanical ventilation. Anesthesiology.

[4] Teboul JL, Monnet X, Chemla D, Michard F (2019). Arterial pulse pressure variation with mechanical ventilation. Am J Respir Crit Care Med.

[5] Michard F, Chemla D, Richard C (1999). Clinical use of respiratory changes in arterial pulse pressure to monitor the hemodynamic effects of PEEP. Am J Respir Crit Care Med.

[6] LAS Vegas investigators (2017). Epidemiology, practice of ventilation and outcome for patients at increased risk of postoperative pulmonary complications: LAS VEGAS – an observational study in 29 countries. Eur J Anaesthesiol.

[7] Levin MA, McCormick PJ, Lin HM (2014). Low intraoperative tidal volume ventilation with minimal PEEP is associated with increased mortality. Br J Anaesth.

[8] Mallat J, Meddour M, Durville E (2015). Decrease in pulse pressure and stroke volume variation after mini-fluid challenge accurately predicts fluid responsiveness. Br J Anaesth.

[9] Myatra SN, Monnet X, Teboul JL (2017). Use of tidal volume challenge to improve the reliability of pulse pressure variation. Crit Care.

[10] Benes J, Giglio M, Brienza N, Michard F (2014). The effects of goal-directed fluid therapy based on dynamic parameters on post-surgical outcome: a meta-analysis of randomized controlled trials. Crit Care.

